# Primary diffuse large B‐cell lymphoma of the bone mimicking osteomyelitis

**DOI:** 10.1002/ccr3.4724

**Published:** 2021-08-30

**Authors:** Abbas Mofidi, Mohsen Esfandbod, Ehsan Pendar, Masoud Mortezazadeh, Alireza Hadizadeh

**Affiliations:** ^1^ School of Medicine Iran university of Medical Sciences Tehran Iran; ^2^ Hematology‐Oncology Department Sina Hospital Tehran University of Medical Sciences Tehran Iran; ^3^ Orthopedic Surgery Department Sina Hospital Tehran University of Medical sciences Tehran Iran; ^4^ Internal Medicine Department Sina Hospital Tehran University of Medical Sciences Tehran Iran; ^5^ School of Medicine Tehran University of Medical Sciences Tehran Iran

**Keywords:** diffuse large B‐cell lymphoma, osteomyelitis, primary bone lymphoma

## Abstract

Consider primary bone lymphoma as an important differential diagnosis of osteomyelitis.In patients who are unresponsive to antibiotics and core needle biopsy result is incoherent with clinical symptoms, consider open biopsy for accurate diagnosis.

## Graphical abstract

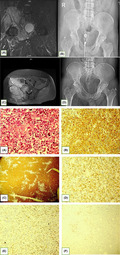

## 1 INTRODUCTION

In this article, we report a 34‐year‐old man who presented with progressive hip pain and osteolytic bone lesions.

Primary workups included core needle biopsies manifested as osteomyelitis; however, as no sign of remission was observed, an open biopsy was considered which revealed primary bone lymphoma.

Primary bone diffuse large B‐cell lymphoma (PB‐DLBCL) is a destructive primary extranodal lymphoma that accounts for 55.7% of all primary bone lymphomas.^1^

Patients are clinically presented with bone pain that is not relieved by rest, soft tissue swelling, palpable mass, pathologic fracture, joint contracture, cord compression, and systemic symptoms such as fever, unintentional weight loss, and night sweats.^2^

The common primary site of bone involvement is the axial skeleton rather than the appendicular skeleton (63 versus 37 percent). ^3^

The diagnosis of (PB‐DLBCL) is based on both imaging and histopathologic findings. Tissue samples can be taken either percutaneously or via open biopsy.

Few primary bone lymphoma cases have been reported in the literature. These cases are usually misdiagnosed, particularly with osteomyelitis. The symptoms often persist for many months before the patient seeks appropriate medical care.^4^

Herein, we describe a 34‐year‐old young man with persistent bone pain whose core needle biopsies were consistent with osteomyelitis; however, his open biopsy revealed PB‐DLBCL to be the underlying cause.

## 2 CASE PRESENTATION

A 34‐year‐old man with no significant past medical or surgical history complaining of dull low back pain and non‐traumatic progressive right hip pain, which he had for 6 months was referred to our clinic. He described his pain as dull low back pain, and he also localized his hip pain on the anterior part of the right hip. The pain also radiated to the buttock and groin. His pain was also exacerbated by weight‐bearing activities and did not relieve even with rest or pain killers, such as morphine. The patient had been bedridden for a month as the pain had gotten excruciating. Meanwhile, the patient had no constitutional symptoms.

The examination revealed decreased range of motion on his right hip joint and tenderness over the anterior joint line, adjacent soft tissue to the right hip joint was also swollen and asymmetric compared to the left side. Other physical exams were unremarkable.

In the previous workup and admission 6 months before referral, the Magnetic resonance imaging (MRI) had demonstrated diffuse abnormal high‐intensity signals on T2 and proton density fat saturation in the right iliac and superior ramus of pubis was seen. This was accompanied by a high‐intensity signal on the peripheral soft tissue along with a periosteal reaction.

Abnormal high‐intensity signals in the medulla of the femur and sequestration in the iliac bone were also reported.

The imaging findings were suggestive for infiltrative disorders. A core needle biopsy under CT scan guidance was obtained from the lesion in the right acetabulum. The pathologic assessment reported unremarkable spongy bone with cellular marrow tissue and a benign lymphocytic aggregation.

These findings were suggestive of osteomyelitis, and subsequently, he was treated with antibiotics, which turned out to be ineffective.

**FIGURE 1:**
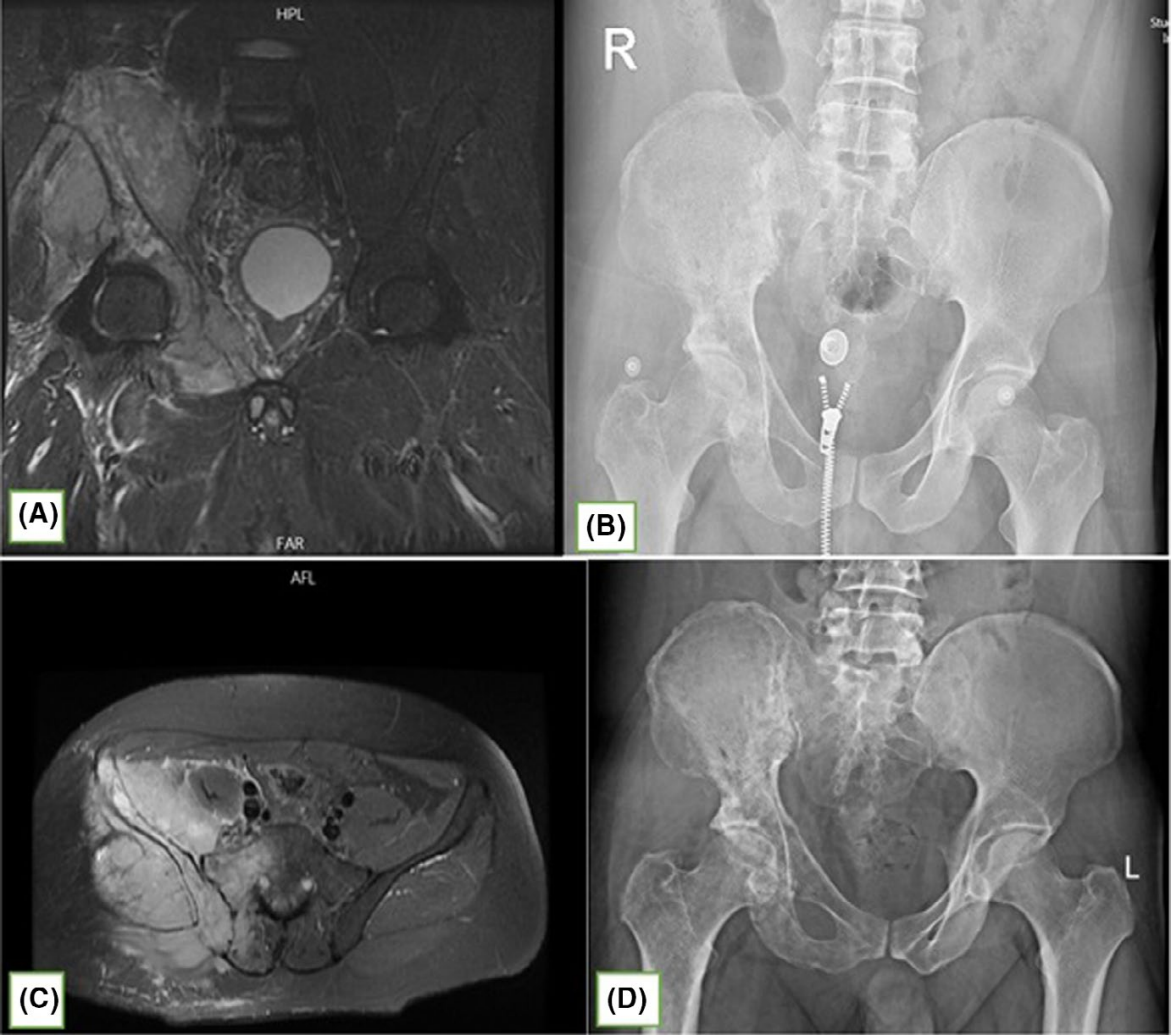
(B &D): X‐ray images of the pelvis that demonstrate lytic radiolucent lesion with destructive pattern and sclerotic areas, (image D taken 4 months later from B), A & C T2‐weighted images with fat saturation, showing high signal intensity in the right iliac and superior ramus of pubis, abnormal high signal intensity in the medulla of femur and sequestration in iliac bone is also seen

Three months after the first admission as the pain had progressed, he has admitted again for further evaluation and the imaging revealed progression of the lesion. (Figure 1).

He became a candidate for another biopsy that showed inflamed fibro connective tissue and fibrin exudative substances, which were compatible with osteomyelitis. He was treated again with broad‐spectrum antibiotics for another 6 weeks.

As the patient's overall health and symptoms showed no sign of remission, he was referred to our clinic for further evaluation. He was admitted and samples were taken. Laboratory results showed the following: white blood cell (WBC) count 5700/µl (band + segment 59.0%, mono 10.0%, lymph 28.0%); hemoglobin (Hgb) 11.5 g/dl; platelet count (Plt) 2.83 × 10^4^/µl; lactate dehydrogenase (LDH) 355 IU/L (normal range 150–500 U/L); AST 13 IU/L; ALT 16 IU/L; and C‐reactive protein (CRP) 25 mg/dl; (ESR) 32 mm/h.

**FIGURE 2:**
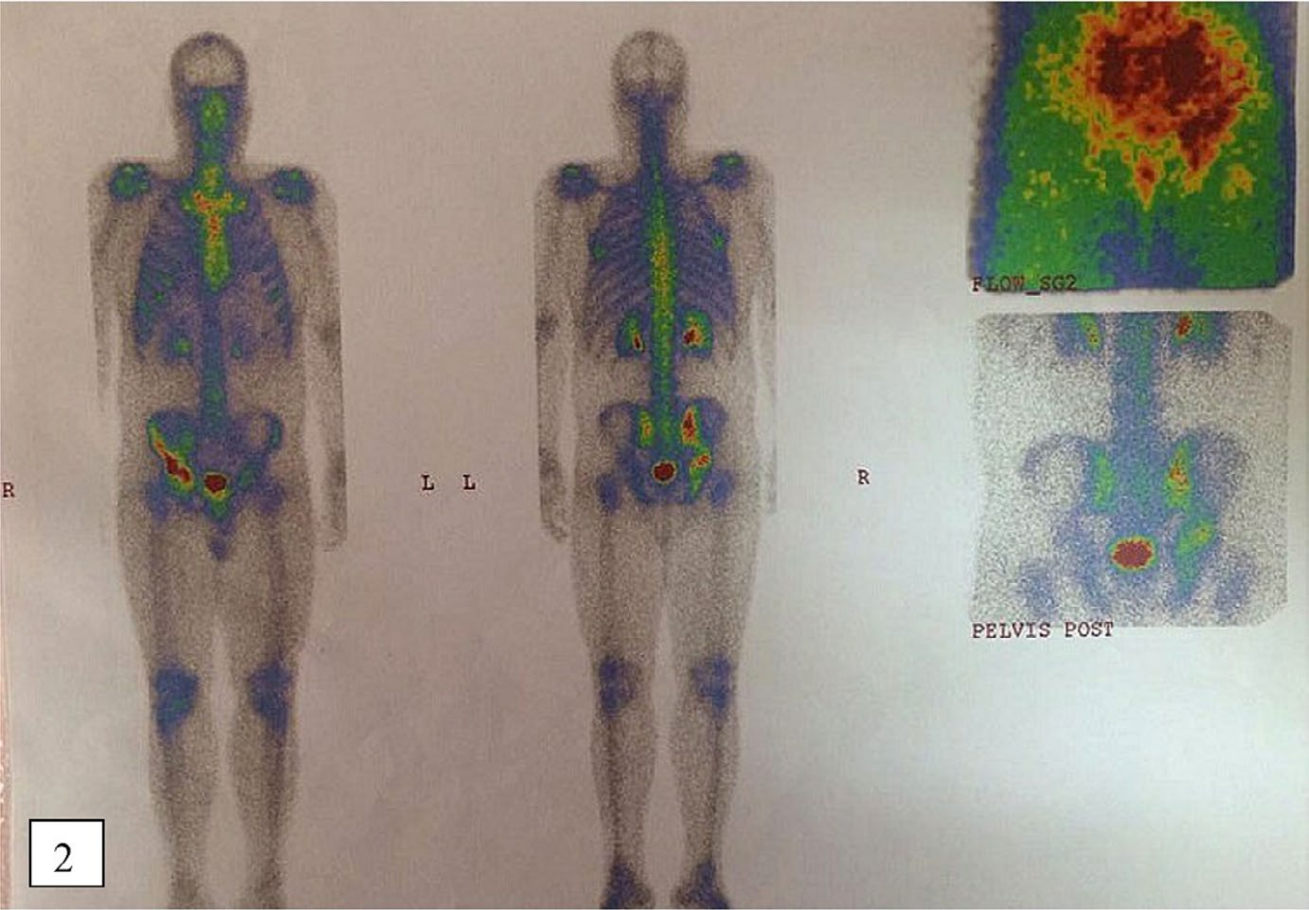
The scan showed moderately to severely increased radiotracer uptake in the right sacroiliac joint, right acetabular joint, and iliac crest

For evaluating the lesions, a whole bone scan with TC‐99m was performed, which was suggestive for chronic arthritis and osteomyelitis in the sacroiliac joint. Right acetabular bone especially in the acetabular roof and the right iliac crest were also suggestive for probable bone tumors. (Figure 2).

According to the clinical findings and the fact that previous core needle biopsies were inconclusive and unhelpful, we decided to perform an open biopsy to rule out primary bone malignancies.

The open biopsy of the lesion and the pathologic assessment suggested primary bone DLBCL.

**FIGURE 3:**
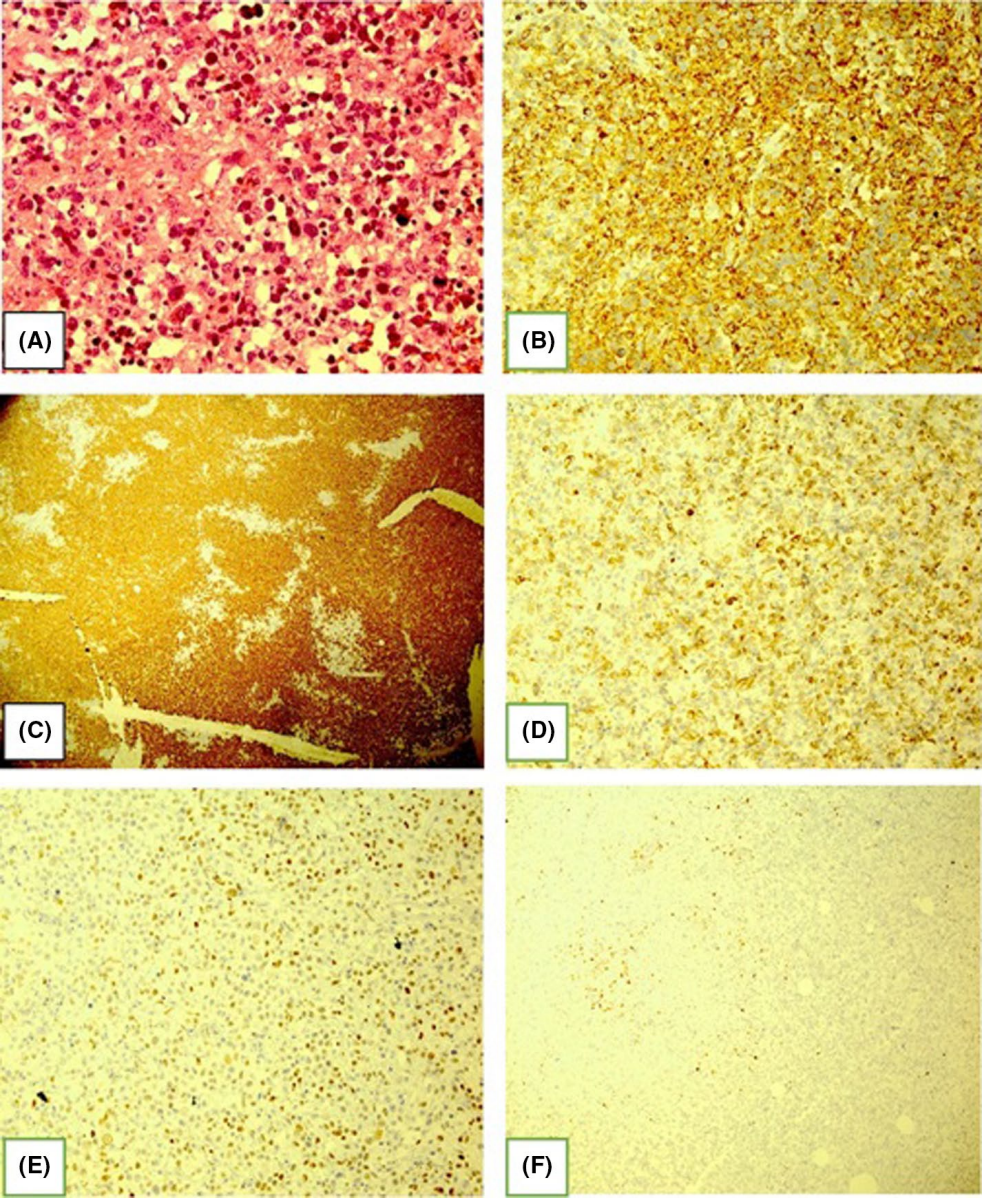
Pathology and IHC of primary bone diffuse large B‐cell lymphoma, non‐GCB, (A) H&E, (B) LCA, (C) CD20 (D) bcl2 (E) bcl6,(F) MUM‐1+

Immunohistochemical results were as following LCA+, CD20+, Bcl‐6+, Bcl 2+, CD3‐, MUM‐1+, CD99‐, vimentin‐, CD10‐, and Ki67+ (90%), this was consistent with DLBCL, non‐germinal center B‐cell like (non‐GCB; Figure 3).

A whole body bone and CT scan, and bone marrow biopsy from the iliac crest, from the opposite side of the lesion, were performed for the staging.

CT scan showed diffuse lytic lesions in the ilium, sacrum, pubis, ischium, and femoral head and neck on the right side.

Visceral involvement or pathologic para aortic lymphadenopathy was not reported.

Using the Ann Arbor staging, the patient was staged at the I EA level of the disease with no other distant metastasis.

And according to International Prognostic Index for Diffuse Large B‐cell Lymphoma, the patient was scored 1 and the predicted 4‐year progression‐free survival rate was estimated at 80%.

The patient received his first cycle of chemotherapy with rituximab, cyclophosphamide, Adriamycin, vincristine, prednisone (R‐CHOP), and concurrent intrathecal chemotherapy with Cytarabin, methotrexate, and hydrocortisone. Meanwhile, he also became a candidate for radiotherapy (RT).

## 3 DISCUSSION

Primary bone lymphoma is a rare extranodal lymphoma, which accounts for 2% of all lymphomas and about 5% of bone tumors in adults.

The majority of patients, pathologically diagnosed as diffuse large B‐cell lymphoma. Other subtypes found in a minority of cases include follicular lymphoma, small lymphocytic lymphoma, marginal zone lymphoma, anaplastic large cell, and Burkitt's lymphoma.

It commonly develops in patients aged between 20 and 50 years, and the most common symptom is bone pain.

In this article, we report a 34‐year‐old man who presented progressive hip pain and osteolytic bone lesions. He was later diagnosed with primary diffuse large B‐cell bone lymphoma, and previous studies had also suggested that osteomyelitis unusually mimics PB‐DLBCL^5^, ^6^, ^7^

Several rationales led us to believe that the previous workup session was insufficient, and another approach is needed to be taken. Hence, we decided to perform an open biopsy. One of the main reasons to do so was the fact that bone pain was progressive and unresponsive to the standard antibiotic regimen.

On the contrary, radiologic changes, such as osteolytic lesions, were progressive and despite regular treatment for osteomyelitis the lesions progressed and contained sequestrum as the disease spread. This pattern of progress was also observed between the two MRI scans that were taken in the 6 months. These changes include an extraosseous extension of the lesion that had led to asymmetric soft tissue swelling. These patterns of change are usually seen with neoplastic lesions rather than infectious ones.

In recent studies, it has been suggested that An ESR level of 60 mm/h is an optimal cutoff level for suspected osteomyelitis (74% sensitive and 56% specific). Our patient had ESR levels below the threshold; thus, osteomyelitis seemed unlikely.^8^

Other reasons such as lack of predisposing factors for osteomyelitis (ie, long term skin infections, prosthetic joints, use of intravenous drugs, etc.) and abrupt beginning of the symptoms made our case less probable for osteomyelitis. Being safer and almost equally accurate, it was suggested by the literature that a core needle biopsy is a more preferable method of obtaining bone tissue samples than an open biopsy. It is also both less invasive and cost‐effective.^9^

Unfortunately, though in our case core needle biopsy could not help provide an accurate diagnosis.

We have come to conclude that the first two biopsies were taken from the rim of the lesion, thus making the sample redundant and inefficient. The result was only suggestive for non‐specific lymphocyte aggregation. In such circumstances, open biopsy seems suitable for patients who are highly suspicious of bone tumors and core needle biopsy is inconclusive and non‐diagnostic.^10^

## 4 CONCLUSION

In brief, our case illustrates the difficulties in distinguishing primary bone lymphoma from osteomyelitis, as both have similar presentations, while the radiologic findings can overlap and mimic each other.

Our experience with this case emphasizes the fact that for an accurate diagnosis, while the clinical manifestations and paraclinical findings are suggestive for malignancies and tumors (in this case lymphoma), the results from core needle biopsy are incoherent, and open biopsy should be considered.

## CONFLICT OF INTEREST

None declared.

## AUTHOR CONTRIBUTIONS

A.M. and M.M. contributed to data collection, writing, drafting of the manuscript, and critical appraisal of the manuscript. A.H, EP, and M.E. contributed to scientific writing and final revision.

## ETHICAL APPROVAL

In this study, no additional costs and procedures were imposed on the patient. We reported the retrograde standard treatment process of the patient. We maintained the patient's privacy, and her written consent was obtained.

## CONSENT TO PARTICIPATE

The patient has consented to the participation of this case report.

## CONSENT FOR PUBLICATION

The participant has consented to the publication of this case report.

## Data Availability

The data that support the findings of this study are available from the corresponding author, [MM], upon reasonable request.

## References

[1] Zhang X, Zhu J, Song Y, Ping L, Zheng W. Clinical characterization and outcome of primary bone lymphoma: a retrospective study of 61 Chinese patients. Sci Rep. 2016;6(1):28834. 27357354 10.1038/srep28834 PMC4928085

[2] Choi JY, Hahn JS, Suh CO, Yang WI. Primary lymphoma of bone–survival and prognosis. Korean J Intern Med. 2002;17(3):191‐197. 12298430 10.3904/kjim.2002.17.3.191 PMC4531681

[3] Jawad MU, Schneiderbauer MM, Min ES, Cheung MC, Koniaris LG, Scully SP. Primary lymphoma of bone in adult patients. Cancer. 2010;116(4):871‐879. 20043324 10.1002/cncr.24828

[4] Mika J, Schleicher I, Gerlach U, Adler CP, Uhl M, Knoeller SM. Primary bone lymphomas thought to be osteomyelitis urgently demand a rapid diagnosis in bone pathology. Anticancer Res. 2012;32(11):4905‐4912. 23155259

[5] Hsieh TC, Kao CH, Yen KY, Sun SS. Osteomyelitis‐mimicking primary bone lymphoma at the hip prosthetic site. Clin Nucl Med. 2007;32(7):543‐544. 17581341 10.1097/RLU.0b013e318065a9cf

[6] Badam T, Devadoss S, Balaji M, Devadoss A, Pn R. Primary Bone Lymphoma Mimicking as Osteomyelitis: An Unusual Presentation. J Orthop Oncol. 2017;02. 10.4172/2472-016X.1000111

[7] Mika J, Schleicher I, Gerlach U, Adler C‐P, Uhl M, Knoeller SM. Primary bone lymphomas thought to be osteomyelitis urgently demand a rapid diagnosis in bone pathology. Anticancer Res. 2012;32(11):4905. 23155259

[8] Lavery LA, Ahn J, Ryan EC, et al. What are the optimal cutoff values for ESR and CRP to diagnose osteomyelitis in patients with diabetes‐related foot infections? Clin Orthop Relat Res. 2019;477(7):1594‐1602. 31268423 10.1097/CORR.0000000000000718 PMC6999976

[9] Pohlig F, Kirchhoff C, Lenze U, et al. Percutaneous core needle biopsy versus open biopsy in diagnostics of bone and soft tissue sarcoma: a retrospective study. Eur J Med Res. 2012;17(1):29. 23114293 10.1186/2047-783X-17-29 PMC3507842

[10] Majeed A, Chan O, Okolo O, Shponka V, Georgescu A, Persky D. Hodgkin lymphoma mimicking osteomyelitis. Case Rep Oncol. 2017;10(2):542‐547. 28690530 10.1159/000474938 PMC5498972

